# Homocitrate Synthase Genes of Two Wide-Host-Range *Bradyrhizobium* Strains are Differently Required for Symbiosis Depending on Host Plants

**DOI:** 10.1264/jsme2.ME19078

**Published:** 2019-12-27

**Authors:** Shun Hashimoto, Jenjira Wongdee, Pongpan Songwattana, Teerana Greetatorn, Kohki Goto, Panlada Tittabutr, Nantakorn Boonkerd, Neung Teaumroong, Toshiki Uchiumi

**Affiliations:** 1 Graduate School of Science and Engineering, Kagoshima University 1–21–35 Korimoto, Kagoshima, Kagoshima 890–0065 Japan; 2 School of Biotechnology, Institute of Agricultural Technology, Suranaree University of Technology 111 University Avenue, Suranaree, Muang, Nakhon Ratchasima 30000 Thailand

**Keywords:** *Bradyrhizobium*, legumes, symbiotic nitrogen fixation, *nifV*, homocitrate

## Abstract

The *nifV* gene encodes homocitrate synthase, the enzyme that catalyzes the formation of homocitrate, which is essential for arranging the FeMo-cofactor in the catalytic center of nitrogenase. Some host plants, such as *Lotus japonicus*, supply homocitrate to their symbionts, in this case, *Mesorhizobium loti*, which lacks *nifV*. In contrast, *Bradyrhizobium* ORS285, a symbiont of *Aeschynomene* cross-inoculation (CI) groups 2 and 3, requires *nifV* for symbiosis with *Aeschynomene* species that belong to CI group 3, and some species belonging to CI group 2. However, it currently remains unclear whether rhizobial *nifV* is required for symbiosis with *Aeschynomene* species belonging to CI group 1 or with other legumes. We generated *nifV*-disruption (Δ*nifV*) mutants of two wide-host-range rhizobia, *Bradyrhizobium* SUTN9-2 and DOA9, to investigate whether they require *nifV* for symbiosis. Both Δ*nifV* mutant strains showed significantly less nitrogenase activity in a free-living state than the respective wild-type strains. The symbiotic phenotypes of SUTN9-2, DOA9, and their Δ*nifV* mutants were examined with four legumes, *Aeschynomene americana*, *Stylosanthes hamata*, *Indigofera tinctoria*, and *Desmodium tortuosum*. *nifV* was required for the efficient symbiosis of SUTN9-2 with *A. americana* (CI group 1), but not for that of DOA9. SUTN9-2 established symbiosis with all three other legumes; *nifV* was required for symbiosis with *I. tinctoria* and *D. tortuosum*. These results suggest that, in addition to *Aeschynomene* CI groups 2 and 3, CI group 1 and several other legumes require the rhizobial *nifV* for symbiosis.

Symbiosis between legumes and rhizobia for nitrogen fixation has been extensively studied for its potential contribution to sustainable agriculture. Rhizobia produce a nodulation factor (Nod-factor, NF) that is known to be important for establishing symbiosis with the host leguminous plant (12). NF is a lipochitooligosaccharide, and its sugar length and modifications are specific to rhizobial species (9). When host plants recognize a NF produced by a compatible rhizobium, they initiate a nodulation program (2, 20, 21). Although photosynthetic *Bradyrhizobium* strains lack canonical *nod*-genes for NF biosynthesis, they are able to establish symbiotic nitrogen fixation with *Aeschynomene* plants, which is independent of NF (8).

The genus *Aeschynomene* comprises approximately 180 species, which are classified into three cross-inoculation (CI) groups according to their symbiotic relationship with *Bradyrhizobium* strains (1, 3, 8, 16). Members of CI group 1 are nodulated by non-photosynthetic *Bradyrhizobium* spp. only in a NF-dependent manner (3, 16). Members of CI group 2 are nodulated by both non-photosynthetic and photosynthetic *Bradyrhizobium* spp. in a NF-dependent manner (3, 16). Members of CI group 3 are nodulated by both non-photosynthetic and photosynthetic *Bradyrhizobium* spp. in a NF-independent manner (3, 16), and are called NF-independent *Aeschynomene* species (3, 8, 16).

Rhizobia produce a molybdenum nitrogenase for nitrogen fixation. This nitrogenase consists of component 1 (MoFe protein) and component 2 (Fe protein). Component 1 is a heterodimer of NifD and NifK, and contains an iron–molybdenum cofactor (FeMo-co) and P-cluster. Component 2 is a homodimer of NifH and contains an iron–sulfur cluster. Other *nif* genes related to the biosynthesis and assembly of the FeMo-co, P-cluster, and iron–sulfur cluster are also required for nitrogenase maturation. One of the nitrogenase maturation genes, *nifV*, encodes homocitrate synthase, which catalyzes the condensation of acetyl-CoA and 2-oxoglutarate; its product, homocitrate, is essential for the positioning of FeMo-co in the catalytic center of nitrogenase (11).

*Mesorhizobium loti*, which lacks *nifV*, utilizes homocitrate supplied by its host plant, *Lotus japonicus*, during symbiosis (10). However, some rhizobia, such as *Bradyrhizobium* ORS285, *Bradyrhizobium* ORS278, and *Azorhizobium caulinodans*, which have their own *nifV*, may fix nitrogen in their free-living state (25). Strain ORS285, a symbiont of *Aeschynomene* CI groups 2 and 3, requires the *nifV* gene for nitrogen fixation in both free-living and symbiotic states with NF-independent *Aeschynomene* species (CI group 3) (18). To establish symbiosis with NF-dependent *Aeschynomene* species (CI group 2), the ORS285 requirement for *nifV* depends on the host plant (18). However, in other rhizobia/legume symbioses, including those with plants in CI group 1, it currently remains unclear whether rhizobial *nifV* is required.

The non-photosynthetic *Bradyrhizobium* strains SUTN9-2 and DOA9 have been isolated from rice fields using *A. americana* (CI group 1) as a trap plant (17, 23). These strains have a wide host range and nodulate on not only *A. americana*, but also on several legume species in other genera (17). Genome sequences are available (17, 19, 24) and both strains possess *nifV*. In the present study, we generated *nifV*-disrupted (Δ*nifV*) mutants of both SUTN9-2 and DOA9 to investigate whether *nifV* is required for nitrogenase activity in both the free-living and symbiotic states. As host plants, we selected *A. americana* and *Stylosanthes hamata* (Dalbergioid clade, the ancestral clade of the Phaseoloid clade), as well as *Indigofera tinctoria* and *Desmodium tortuosum* (Phaseoloid clade, the ancestral clade of the Robinioids clade to which *L. japonicus* belongs). In the present study, we show that rhizobial *nifV* was differently required for symbiotic nitrogen fixation in a manner that depended on rhizobium–host plant pairing and the age of the nodule.

## Materials and Methods

### Bacterial strains and growth conditions

The bacterial strains and plasmids used in the present study are listed in Table 1. *Bradyrhizobium* SUTN9-2 and DOA9 and their derivatives were grown at 28°C in yeast-mannitol (7), BNM-B (22), or HEPES-MES salt medium (4) supplemented with arabinose (0.1% [w/v]) and yeast extract (0.25% [w/v]). *Escherichia coli* strains were grown at 37°C in Luria–Bertani medium. When required, each medium was supplemented with the following reagents and/or antibiotics for the construction of *nifV* mutant strains: sucrose (10% [w/v]), cefotaxime (20 μg mL^−1^), kanamycin (50 μg mL^−1^), or gentamicin (50 μg mL^−1^).

### Phylogenetic tree of NifV proteins and map of *nifV* genes

The amino acid sequences of all rhizobial NifV proteins were collected from MicroScope (24) (microbe genome database, http://www.genoscope.cns.fr/agc/microscope) using homocitrate synthase as the keyword. To collect rhizobial NifV proteins, BLASTP was also performed with NifV of SUTN9-2 as a query sequence. A neighbor-joining phylogenetic tree was generated from a ClustalW2 alignment, with *Azotobacter vinelandii* NifV as the outgroup. Genetic mapping of the region that includes *nifV* was also performed in MicroScope for both strains.

### Construction of *nifV* mutant strains

To construct *nifV*-lacking mutants of *Bradyrhizobium* SUTN9-2 and DOA9, the regions 1 kb upstream and downstream of the *nifV* ORF were amplified individually by PCR using the following primer set: up.nifV.BamHI.SUTN9-2.f: 5′-ATGCCGGGATCC CGACCGACAGCAATCTCGAT-3′, up.nifV.HindIII.SUTN9-2.r: 5′-CGAAAGCTGGAAGCTTGCAAGAGCTACTCATTGTTTGA CCTAC-3′, dw.nifV.HindIII.SUTN9-2.f: 5′-TAGCTCTTGCAAGC TTCCAGCTTTCGCACGTCAGATC-3′, dw.nifV.XbaI.SUTN9-2.r: 5′-CCTCGAATCTAGAGCGCGGGTCTCAGCAGGTCGTAA-3′, up.nifV.BamHI.DOA9.f: 5′-TGCACCGGATCCCCTTGCACGCTT CTGCAAT-3′, up.nifV.HindIII.DOA9.r: 5′-ATTGGAGGACGCAAG CTTGGGTATCATGGCCTGCATCGT-3′, dw.nifV.HindIII.DOA9.f: 5′-GCCATGATACCCAAGCTTGCGTCCTCCAATCATTCATTC-3′, dw.nifV.XbaI.DOA9.r: 5′-ACGACGTCTAGATGCACGGATTGC AACGATTTC-3′. Upstream and downstream DNA fragments were connected by crossover PCR and ligated into the suicide plasmid pNPTS129 (Table 1) containing the *sacB* marker using the restriction enzymes *Bam*HI and *Xba*I. A gene for cefotaxime resistance was inserted between the upstream and downstream regions using *Hin*dIII. The resulting plasmids pNPTS129/9-2*nifV*UP/*cefotaxime^r^*/9-2*nifV*DW (Table 1) and pNPTS129/DOA9*nifV*UP/*cefotaxime^r^*/DOA9*nifV*DW (Table 1) were transformed into *E. coli* DH5α and then transferred into SUTN9-2 and DOA9 individually by tri-parental mating using *E. coli* carrying pRK2013 (Table 1) (6) as a helper. The *nifV* gene was mutated by homologous recombination. *nifV* mutant candidates were selected on HM agar plates containing cefotaxime (20 μg mL^−1^) and sucrose (10% [w/v]). These candidates were verified by PCR, and the strains in which the *nifV* gene was replaced with the cefotaxime resistance gene are referred to as *nifV*-disrupted (Δ*nifV*) mutant strains.

### Nitrogenase activity under free-living conditions

To estimate the nitrogenase activity of *Bradyrhizobium* SUTN9-2, DOA9, and their Δ*nifV* mutants under free-living conditions, *Bradyrhizobium* strains grown in yeast-mannitol liquid medium were washed with BNM-B medium and resuspended in BNM-B soft agar (0.8% [w/v]) medium at OD_600_=0.15. These suspensions (2 mL) were transferred into 10-mL test tubes (BD Vacutainer, Franklin Lakes, NJ, USA). The tubes were capped, and 0.8 mL of the air in the test tube was replaced with acetylene. After an incubation (at 28°C for 7 d), ethylene, as the product of the acetylene reduction activity (ARA) of nitrogenase, was measured by gas chromatography. In complementation studies, homocitrate was added to the BNM-B soft agar medium at a final concentration of 1 mM.

### Plant growth and symbiotic phenotype analyses

The symbiotic phenotypes of *Bradyrhizobium* SUTN9-2, DOA9, and their Δ*nifV* mutants were analyzed when paired with four leguminous species, *A. americana*, *S. hamata*, *I. tinctoria*, and *D. tortuosum*. The seeds of *A. americana* were surface-sterilized in conc. sulfuric acid for 30 min and washed with sterilized water. Seeds of *S. hamata* were surface-sterilized in 3% sodium hypochlorite and 0.1% Tween 20 for 5 min and then washed with sterilized water. Seeds of *I. tinctoria* and *D. tortuosum* were surface-sterilized in conc. sulfuric acid for 10 min and 0.2% sodium hypochlorite and 0.1% Tween 20 for 40 min and then washed with sterilized water. After surface sterilization, the seeds were transferred onto 0.8% agar plates and germinated at 28°C. Two-day-old seedlings were transferred to the top of a test tube containing vermiculite and BNM (5) liquid medium (BNM liquid medium without vermiculite was used for *S. hamata*) and grown at 28°C with a 12-h light/dark cycle. After 1 week, each seedling was inoculated with 1 mL of a rhizobial suspension adjusted to an OD_600_=1.0. Plant fresh weight, nodule number, and ARA were measured on day 10 or 21 after the inoculation.

## Results

### Distribution of the *nifV* gene in rhizobia

To assess what percentage of rhizobial strains possess the *nifV* gene, we searched all rhizobia in the MicroScope database using homocitrate synthase as a keyword. We also performed BLASTP with NifV of SUTN9-2 as a query sequence. The keyword search and BLASTP analysis provided the same results. Putative *nifV* genes were identified in 81 out of 148 *Bradyrhizobium* strains (55%), in 3 out of 7 *Mesorhizobium* strains (43%), and in 1 out of 85 *Sinorhizobium* strains (1%) (Table S1). No putative *nifV* genes were identified in any of the 30 *Rhizobium* strains. Overall, 31% of rhizobia in the MicroScope database possess a *nifV* gene.

### *nifV* and its homologues in SUTN9-2 and DOA9

**Fig. 1:**
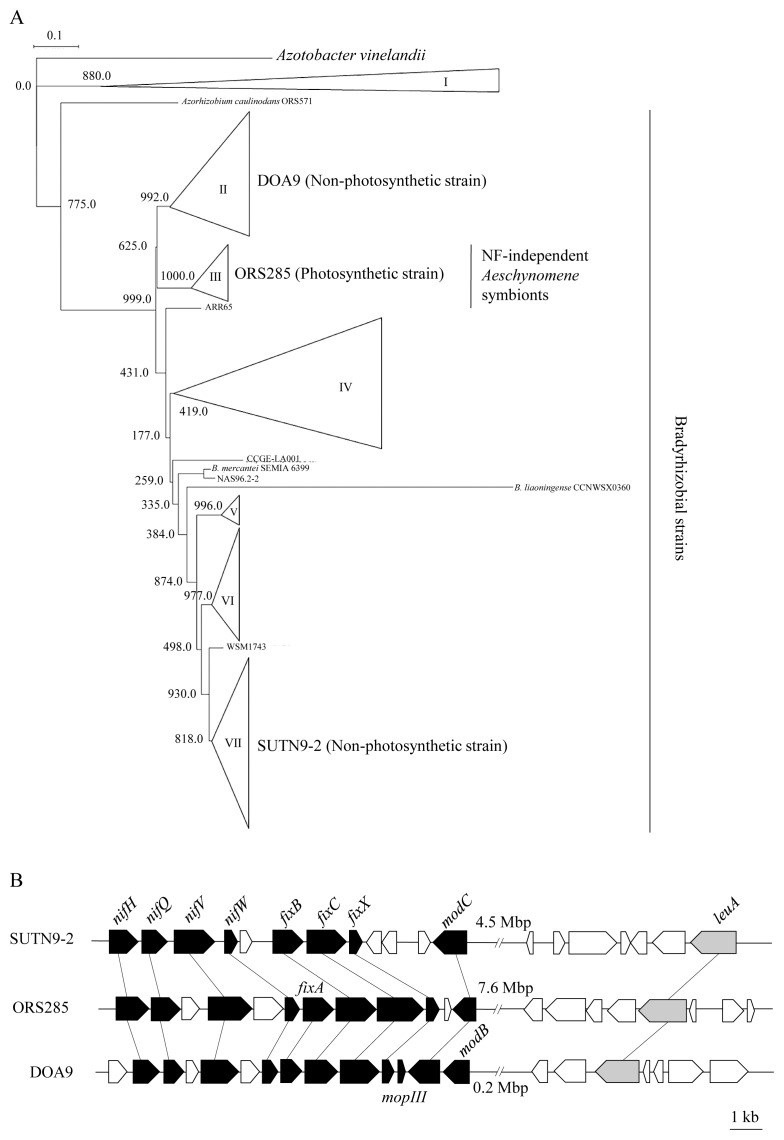
Phylogenetic tree of NifV proteins and genetic map of the *nifV* gene. (A) Neighbor-joining phylogenetic tree of NifV proteins. The bar shows one estimated substitution of an amino acid per 10 amino acid positions. (B) Genetic organization of the *nifV* and *2-isopropylmalate synthase* genes (*leuA*) in *Bradyrhizobium* strains. All bacteria used for the phylogenic tree are listed in supplementary Table S1.

A sequence analysis of genomic DNA revealed that SUTN9-2 and DOA9 each possess a putative *nifV* gene; the encoded proteins show 78 and 81% sequence identities, respectively, with the NifV protein of ORS285. We generated a phylogenetic tree of rhizobial NifV protein sequences collected from MicroScope. The predicted NifV proteins of the SUTN9-2 and DOA9 strains, which are non-photosynthetic, were found to be distinct from those of the symbionts of NF-independent *Aeschynomene* species (clade III) (Fig. 1A). The NifV proteins of strains SUTN9-2 and DOA9 were classified into different clades, each containing other bradyrhizobial NifV (Fig. 1A). The *nifV* genes of SUTN9-2 and DOA9 were both found to be clustered with other *nif* genes (Fig. 1B), as in ORS285. We constructed *nifV*-disruption (Δ*nifV*) mutants derived from the SUTN9-2 and DOA9 strains by in-frame replacing the *nifV* ORF with a cefotaxime-resistant gene. ORS285 contains another gene whose product shares 35% amino acid sequence identity with its own NifV protein. The encoded protein is annotated as 2-isopropylmalate synthase (EC 2.3.3.13) and belongs to the same family as homocitrate synthase (EC 2.3.3.14). SUTN9-2 and DOA9 both carry another gene, annotated as a 2-isopropylmalate synthase (*leuA*); the encoded proteins share 33 and 34% amino acid sequence identities with the respective NifV proteins. In both strains, the *leuA* genes were located outside of the *nif* gene cluster (Fig. 1B).

### Acetylene reduction activity under free-living conditions

**Fig. 2:**
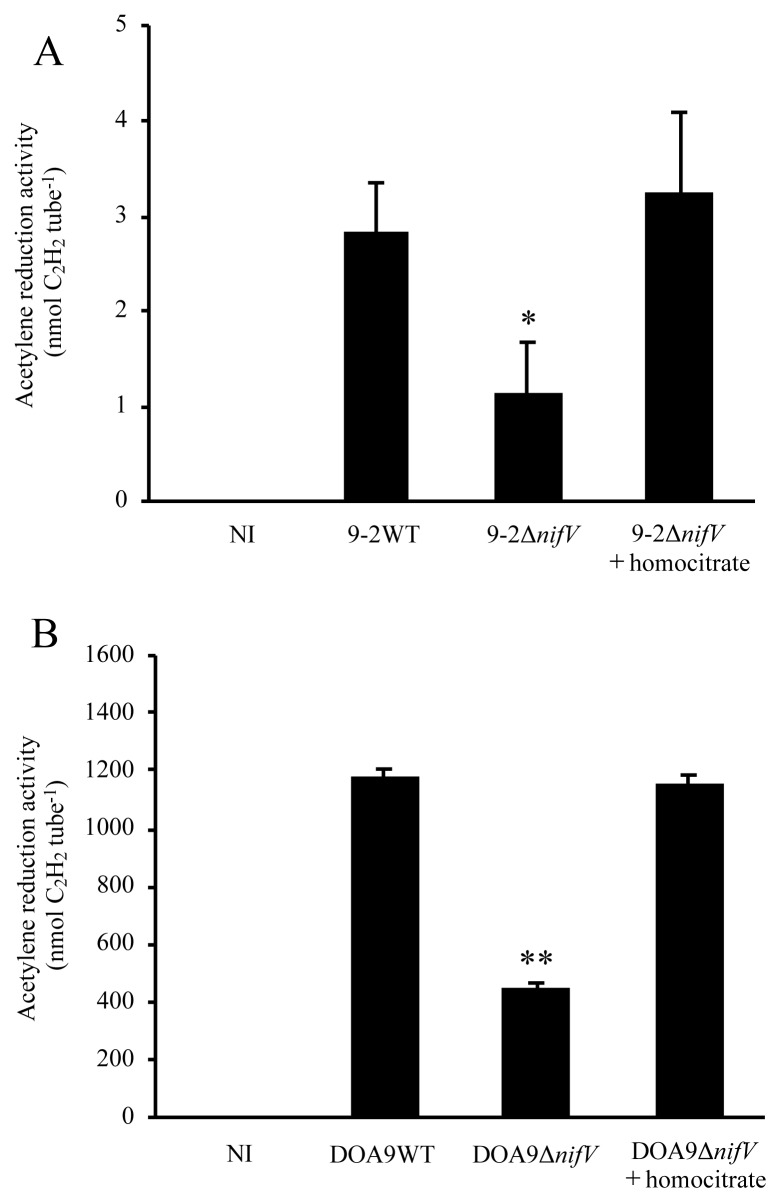
Nitrogenase activity under the free living state of *Bradyrhizobium* strains. Nitrogenase activity was estimated as acetylene reduction activity (ARA). (A) SUTN9-2 and SUTN9-2Δ*nifV*. (B) DOA9 and DOA9Δ*nifV*. Homocitrate was added to growth media at a final concentration of 1 mM. NI, no inoculum as a control. Values are means±SE (*n*=3). Asterisks indicate a significant difference between the wild type and Δ*nifV* mutant (* *P*<0.05, ** *P*<0.01, the Student’s *t*-test).

To verify the function of *nifV* in SUTN9-2 and DOA9, we measured the ARA to estimate the nitrogenase activities of both strains and their Δ*nifV* mutants under free-living conditions (Fig. 2). Both strains showed ARA under free-living conditions, although the ARA of SUTN9-2 was markedly lower than that of DOA9. ARA was significantly lower in the Δ*nifV* strains, but was still detectable in both. Supplementation with exogenous homocitrate restored the ARA of both Δ*nifV* mutants to the levels of their parent strains (Fig. 2).

### Symbiotic phenotypes of Δ*nifV* mutants with four leguminous species

To investigate whether the deletion of *nifV* affects symbiosis, four leguminous species were inoculated with SUTN9-2, DOA9, or their Δ*nifV* mutants.

**Fig. 3:**
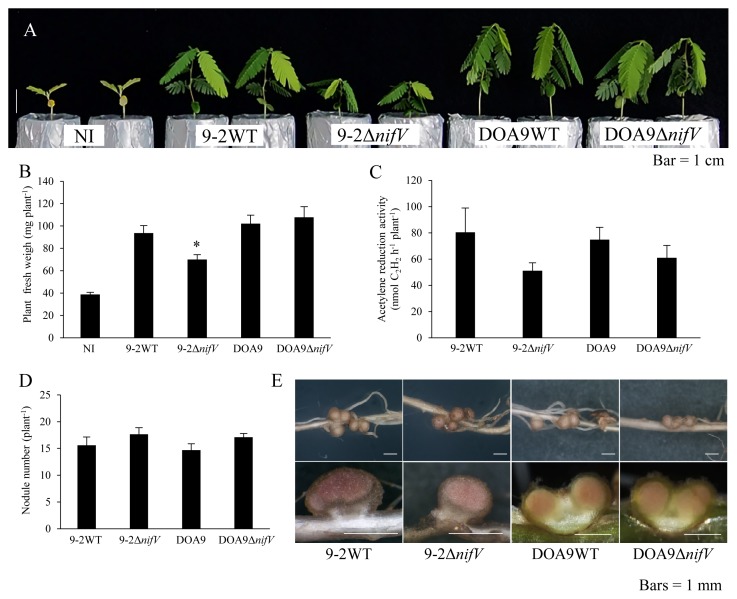
Symbiotic phenotypes of *Bradyrhizobium* strains with *Aeschynomene americana* three weeks after the inoculation. Plant growth (A), plant fresh weight (B), ARA as nitrogenase activity (C), nodule number (D), and nodules and their cross section (E). NI, no inoculum as a control. Values are means±SE (*n*=10), and asterisks indicate a significant difference between the wild type and Δ*nifV* mutant (* *P*<0.01).

In comparisons with *A. americana* plants inoculated with wild-type SUTN9-2, those inoculated with SUTN9-2Δ*nifV* showed significantly decreased plant growth (Fig. 3A and B), and ARA also decreased by 36% (Fig. 3C). These plants also had 13% more nodules (Fig. 3D). Comparisons of the nodules induced by SUTN9-2 and SUTN9-2Δ*nifV* on *A. americana* showed no marked differences, and the appearance of nodule sections did not significantly differ (Fig. 3E). *A. americana* plants inoculated with DOA9Δ*nifV* showed a normal symbiotic phenotype (Fig. 3). No significant differences were detected in plant fresh weights (Fig. 3B).

**Fig. 4:**
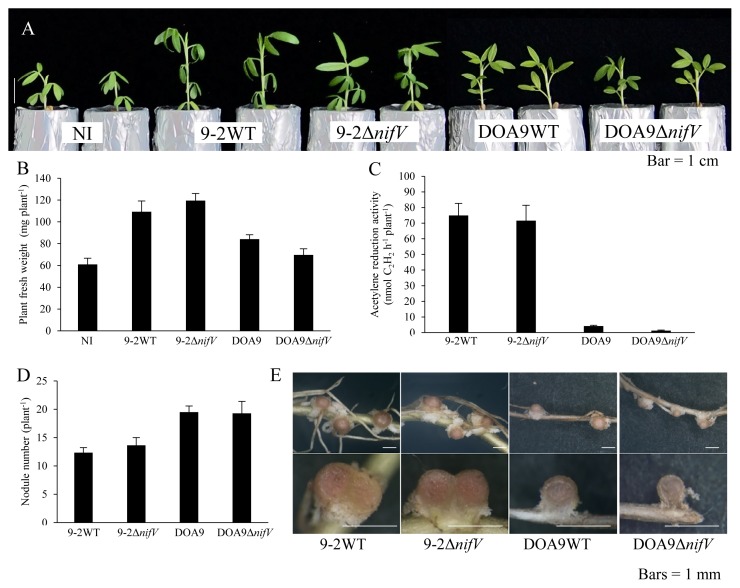
Symbiotic phenotypes of *Bradyrhizobium* strains with *Stylosanthes hamata* three weeks after the inoculation. Plant growth (A), plant fresh weight (B), ARA as nitrogenase activity (C), nodule number (D), and nodules and their cross section (E). NI, no inoculum as a control. Values are means±SE (*n*=10).

*S. hamata* plants inoculated with SUTN9-2Δ*nifV* showed a normal symbiotic phenotype (Fig. 4). An inoculation with DOA9 induced small nodules (Fig. 4E) with very low nitrogenase activity (Fig. 4C). However, the growth of the inoculated plants was 14% better than that of plants without the inoculation (Fig. 4B). No significant differences were observed between plants inoculated with DOA9 and DOA9Δ*nifV* (Fig. 4).

**Fig. 5:**
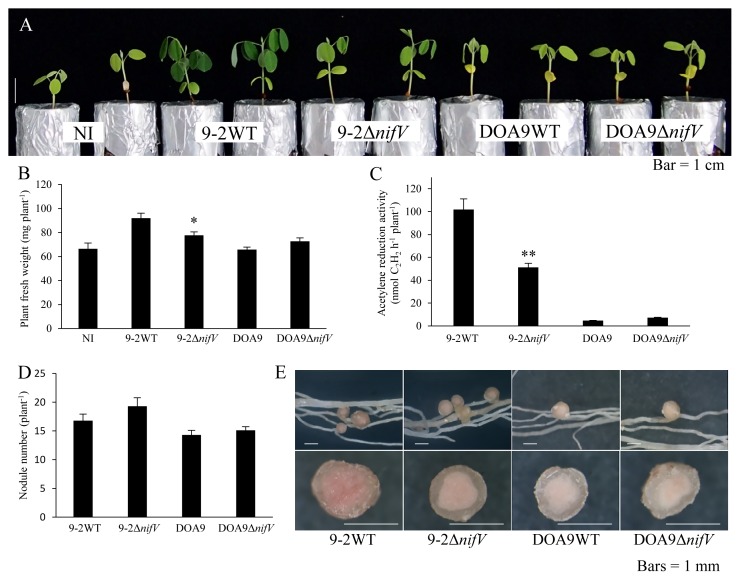
Symbiotic phenotypes of *Bradyrhizobium* strains with *Indigofera tinctoria* three weeks after the inoculation. Plant growth (A), plant fresh weight (B), ARA as nitrogenase activity (C), nodule number (D), and nodules and their cross section (E). NI, no inoculum as a control. Values are means±SE (*n*=10), and asterisks indicate a significant difference between the wild type and Δ*nifV* mutant (* *P*<0.05, ** *P*<0.01, the Student’s *t*-test) (B, C, and D).

*I. tinctoria* plants inoculated with SUTN9-2Δ*nifV* showed more nitrogen starvation (a yellowish color) than plants inoculated with SUTN9-2 (Fig. 5A). Plants inoculated with SUTN9-2Δ*nifV* showed significantly decreased plant fresh weight and ARA (Fig. 5B and C), and 15% more nodules (Fig. 5D). The nodules induced by SUTN9-2Δ*nifV* were paler than those induced by SUTN9-2 (Fig. 5E). DOA9 nodules showed lower ARA and poorer plant growth promotion than SUTN9-2 nodules (Fig. 5A, B, and C). No significant differences were observed between plants inoculated with DOA9 and DOA9Δ*nifV* (Fig. 5).

**Fig. 6:**
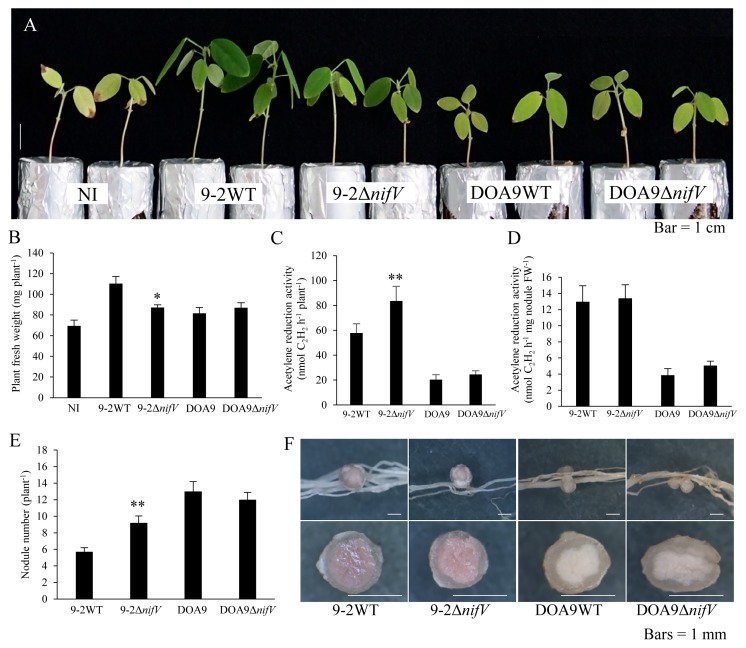
Symbiotic phenotypes of *Bradyrhizobium* strains with *Desmodium tortuosum* three weeks after the inoculation. Plant growth (A), plant fresh weight (B), ARA per plant (C), ARA per nodule fresh weight (FW) (D), nodule number (E), and nodules and their cross section (F). NI, no inoculum as a control. Values are means±SE (*n*=10), and asterisks indicate a significant difference between the wild type and Δ*nifV* mutant (* *P*<0.05, ** *P*<0.01, the Student’s *t*-test).

The growth of *D. tortuosum* plants inoculated with SUTN9-2Δ*nifV* was significantly decreased (Fig. 6A and B), although ARA per plant was significantly higher than in those inoculated with SUTN9-2 (Fig. 6C). The nodules of SUTN9-2 and SUTN9-2Δ*nifV* showed the same ARA on a per-nodule weight (Fig. 6D). The number of nodules induced by SUTN9-2Δ*nifV* was significantly higher than that induced by SUTN9-2 (Fig. 6E). The nodules induced by SUTN9-2Δ*nifV* were pink, the same as the nodules induced by SUTN9-2 (Fig. 6F). Plants of *D. tortuosum* inoculated with DOA9 showed lower ARA and poorer plant growth than those inoculated with SUTN9-2 (Fig. 6A, B, C, and D). No significant differences were observed between plants inoculated with DOA9 and DOA9Δ*nifV* (Fig. 6).

### Symbiotic phenotype of *D. tortuosum* inoculated with SUTN9-2Δ*nifV* on day 10 after the inoculation

**Fig. 7:**
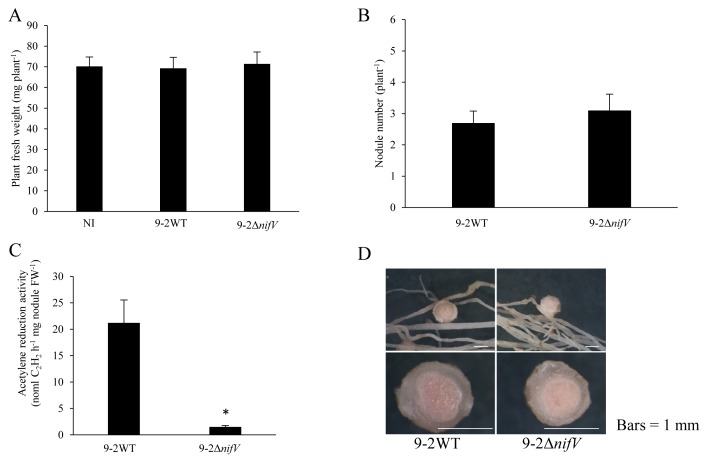
Symbiotic phenotypes of *Bradyrhizobium* SUTN9-2 and SUTN9-2Δ*nifV* with *Desmodium tortuosum* on day 10 after the inoculation. Plant fresh weight (A), nodule number (B), ARA per nodule fresh weight (FW) (C), and nodules and their cross section (D). NI, no inoculum as a control. Values are means±SE (*n*=10), and asterisks indicate a significant difference between the wild type and Δ*nifV* mutant (* *P*<0.01, the Student’s *t*-test).

Since the plants of *D. tortuosum* inoculated with SUTN9-2Δ*nifV* showed significantly decreased plant fresh weight, but unaffected ARA per nodule weight 3 weeks after the inoculation (Fig. 6B and D), we hypothesized that the deletion of *nifV* in SUTN9-2 decreased ARA in younger nodules (10 d after the inoculation) on *D. tortuosum*, resulting in decreased plant growth 3 weeks after the inoculation. To test this hypothesis, we analyzed the symbiotic phenotypes of *D. tortuosum* inoculated with SUTN9-2 and SUTN9-2Δ*nifV* on day 10 after the inoculation. No significant differences were observed in plant fresh weight between plants inoculated with SUTN9-2 and those inoculated with SUTN9-2Δ*nifV*; however, nodule numbers increased by 15% (Fig. 7A and B). The ARA of SUTN9-2Δ*nifV* nodules was significantly lower than that of SUTN9-2 nodules (Fig. 7C). Furthermore, the nodules induced by SUTN9-2Δ*nifV* were paler than those induced by SUTN9-2 (Fig. 7D).

## Discussion

*nifV*, one of the genes related to nitrogenase maturation, encodes homocitrate synthase, which is essential for arranging the FeMo-cofactor in the catalytic center of nitrogenase (11). Database searches for *nifV* revealed that most rhizobia carrying *nifV* are *Bradyrhizobium* strains (Fig. 1A and Table S1). We generated *nifV*-disrupted (Δ*nifV*) mutants of two wide-host-range *Bradyrhizobium* strains, SUTN9-2 and DOA9, to investigate how *nifV* functions in symbiotic nitrogen fixation with four leguminous plants.

We measured the ARA of both strains and their Δ*nifV* mutants under free-living conditions. The Δ*nifV* mutants of both strains showed significantly lower, but still detectable ARA (Fig. 2). Exogenous homocitrate restored the ARA of the Δ*nifV* mutants of both SUTN9-2 and DOA9 (Fig. 2). These results were identical to those reported for a *nifV* deletion mutant of ORS285 (18), suggesting that *nifV* is involved in efficient nitrogenase activity under free-living conditions in both SUTN9-2 and DOA9. We have two hypotheses for why the ARA of these Δ*nifV* mutants did not completely disappear under free-living conditions. The nitrogenase of these Δ*nifV* mutant strains may retain the ability to reduce acetylene to ethylene by using citrate instead of homocitrate at its catalytic center. Although the diazotrophs *Klebsiella pneumoniae* and *Azotobacter vinelandii* use molybdenum nitrogenase, and *nifV* mutants retain ARA at approximately 80 and 10% of the respective wild-type strains under free-living conditions, the nitrogenases of these mutants reduce dinitrogen poorly; 7% of wild-type *K. pneumoniae* and 2% of wild-type *A. vinelandii* (13, 15). A crystallographic analysis of the nitrogenase MoFe protein from the *nifV* mutant of *K. pneumoniae* showed that citrate is a ligand of FeMo-co in the catalytic center (14). Another possibility is that these strains may synthesize a small amount of homocitrate via other proteins. SUTN9-2, DOA9, and ORS285 possess putative genes for 2-isopropylmaleate synthases that show approximately 33, 34, and 35% amino acid sequence identities, respectively, with their own NifV proteins. These genes may encode enzymes that produce homocitrate in place of the NifV protein. Under free-living conditions, the ARA of SUTN9-2 was markedly lower than that of DOA9 (approximately 1/300 to 1/1,000) (Fig. 2) or of other rhizobia that possess a *nifV* gene, such as *Bradyrhizobium* ORS285, ORS278, and *A. caulinodans* ORS571 (25) even though the ARA of SUTN9-2 nodules was as high as that of DOA9 nodules. This suggests that the regulation of *nif* genes in SUTN9-2 under free-living conditions differs from that of other rhizobial strains.

We found that the *nifV* of DOA9 was involved in ARA under free-living conditions, but not in symbiosis with *A. americana* (Fig. 2 and 3). These results suggest that the *nifV* in DOA9 functions for free-living conditions rather than for symbiosis. DOA9 possesses two copies of *nifA* (master regulator of *nif* genes), *nifD* and *nifK*, which are located on the chromosome and on the plasmid, respectively (19, 24–26). These two copies of *nifADK* are functionally redundant for symbiosis with *A. americana*, while the chromosomal *nifADK* is a major contributor for nitrogenase activity under free-living conditions (25, 26). Hence, we infer that the *nifV* of DOA9, which is located on the chromosome, cooperates with chromosomal *nifADK* for nitrogenase activity under free-living conditions. SUTN9-2, not harboring any plasmid, possesses a single copy of *nifA*, *nifD*, and *nifK* on its chromosome (24). We found that the *nifV* of SUTN9-2 was involved in ARA under free-living conditions (Fig. 2). However, SUTN9-2 showed markedly lower ARA (approximately 1/300 to 1/1,000) under free-living conditions than that of DOA9 (Fig. 2). We also noted that the *nifV* of SUTN9-2 contributed to symbiosis with three legumes: *A. americana*, *I. tinctoria*, and *D. turtuosum* (Fig. 3, 4, 5, 6, and 7). These results suggest that the *nifV* of SUTN9-2 functions for symbiosis rather than for free-living conditions in contrast to *nifV* of DOA9.

Plant fresh weight and ARA were lower and nodule numbers were higher in *A. americana*, *I. tinctoria*, and *D. tortuosum* inoculated with SUTN9-2Δ*nifV* than in those inoculated with wild-type SUTN9-2 (Fig. 3, 5, 6, and 7). The low ARA of SUTN9-2Δ*nifV* may affect not only plant growth, but also the number of nodules on host plants. On the other hand, DOA9 showed poorer ARA than SUTN9-2 when associated with *S. hamata*, *I. tinctoria*, and *D. tortuosum* (Fig. 4, 5, and 6). The symbiotic phenotypes of DOA9Δ*nifV* indicate that the deletion of *nifV* did not affect symbiosis with all species in the same manner (Fig. 3, 4, 5, and 6), suggesting that DOA9 is naturally incompatible with these three host plant species.

In the case of *A. americana* (CI group 1), the inoculation with SUTN9-2Δ*nifV* decreased plant growth, whereas that with DOA9Δ*nifV* did not (Fig. 3A and B). These results suggest two possibilities. In DOA9, the homocitrate needed for symbiotic nitrogen fixation with *A. americana* may be synthesized by 2-isopropylmalate synthase instead of by the homocitrate synthase encoded by *nifV*. It is also possible that *A. americana* does not supply sufficient homocitrate for SUTN9-2, but supplies enough for DOA9. In order to distinguish between these two possibilities, it will be necessary to investigate the symbiotic phenotype of DOA9 with the double mutation of *nifV* and *2-isopropylmalate synthase* and to analyze the gene expression of the *homocitrate synthase* (*FEN1* homologue) (10) of *A. americana*. The species of *Aeschynomene* CI group 1 will possess the *FEN1* homologue because these species may establish symbiosis with some *nifV*-lacking strains, such as *B. japonicum* USDA110 (3). However, the *FEN1* homologue of *A. americana* has not yet been identified.

The inoculation with SUTN9-2Δ*nifV* markedly decreased ARA in *D. tortuosum* by day 10 after the inoculation (Fig. 7C). Three weeks after the inoculation, plants inoculated with SUTN9-2Δ*nifV* showed less growth, but higher ARA (Fig. 6B and C). At this time point, the nodule number of plants inoculated with SUTN9-2Δ*nifV* was significantly higher than that of those inoculated with SUTN9-2 (Fig. 6D). This higher nodule number may be attributed to the low ARA on day 10 after the inoculation (Fig. 7C), *i.e.* the low ARA induced the host plant to form more nodules. After the host plant began to supply homocitrate to the newly formed nodules, SUTN9-2Δ*nifV* nodules may have begun to show an ARA at the same level as SUTN9-2 nodules; this may explain the similar ARA levels observed 3 weeks after the inoculation (Fig. 6D). These results suggest that in young nodules (10 d after the inoculation) formed by SUTN9-2 on *D. tortuosum*, *nifV* is required for symbiotic nitrogen fixation. We infer that the low nitrogenase activity of SUTN9-2Δ*nifV* nodules caused the higher nodule number and lower plant growth observed 3 weeks after the inoculation.

A combined summary of the present results and previous findings is shown in Table 2. We found that the inoculation with SUTN9-2Δ*nifV* caused poor plant growth in three out of the four host plants. However, plants inoculated with SUTN9-2Δ*nifV* showed better growth than the non-inoculated control plants of the same species (indicated with + in the light gray cells in Table 2). This pattern is similar to that observed in NF-dependent *Aeschynomene* (CI group 2)/ORS285 symbiosis, but different from NF-independent *Aeschynomene* (CI group 3)/ORS285 symbiosis. In some NF-independent *Aeschynomene* species, an inoculation with ORS285Δ*nifV* resulted in poor plant growth, similar to non-inoculated control plants of the same species (indicated as ++ in the dark gray cell in Table 2) (18). SUTN9-2 has the canonical *nod*-genes to synthesize NF and may establish symbiosis with host plants in a NF-dependent manner. Thus, the present results suggest that the requirement of rhizobial *nifV* for symbiosis is higher in NF-independent symbiosis than in NF-dependent symbiosis, which is consistent with previous findings (18).

We showed that rhizobial *nifV* is required for efficient symbiosis not only with *Aeschynomene* species of CI groups 2 and 3, but also with *A. americana* of CI group 1 and two legumes in the Phaseoloid clade. The symbiotic phenotypes of *A. americana* and *D. tortuosum* suggest that the requirement for rhizobial *nifV* depends on the symbiont–host combination, and also on the age of the nodules. These results contribute to our understanding of the mechanisms contributing to and the evolution of symbiotic nitrogen fixation.

## SUPPLEMENTARY MATERIAL



## Figures and Tables

**Table 1:** Strains and plasmids used in the present study.

Strain or plasmid	Source of isolation or relevant characteristics of the plasmid	Source or reference
**Strains**		
*Bradyrhizibium* spp.		
SUTN9-2	*Aeschynomene americana* nodule	(17)
DOA9	*A. americana* nodule	(17)
SUTN9-2Δ*nifV*	SUTN9-2 derivative, *nifV*::cefotaxime resistance gene; Cefo^r^	This study
DOA9Δ*nifV*	DOA9 derivative, *nifV*::cefotaxime resistance gene; Cefo^r^	This study
*E. coli* DH5α	*recA*; cloning strain	Takara Bio (Kusatsu, Japan)
**Plasmids**		
pNPTS129	*SacB* counterselection vector, Km^r^	Dicon Alley
pNPTS129/9-2*nifV*UP/*cefotaxime^r^*/9-2*nifV*DW	pNPTS129 derivative, carrying the SUTN9-2Δ*nifV* fragment; Km^r^, Cefo^r^	This study
pNPTS129/DOA9*nifV*UP/*cefotaxime^r^*/DOA9*nifV*DW	pNPTS129 derivative, carrying the DOA9Δ*nifV* fragment; Km^r^, Cefo^r^	This study
pRK2013	Co1E1 replicon carrying RK2 transfer genes, Km^r^	(6)

**Table 2:** Requirement of rhizobial *nifV* for each legume/rhizobia symbiosis.

Legume clade	Plant species	*Aeschynomene* CI group	Symbiont rhizobia	Requirement of rhizobial *nifV* for plant growth	Reference
Dalbergioids	*Aeschynomene americana*	CI group1	*Bradyrhizobium* SUTN9-2	+	This study
			*Bradyrhizobium* DOA9	−	
	*A. afraspera*	CI group2	*Bradyrhizobium* ORS285	−	(18)
	*A. nilotica*			+	
	*A. evenia* and six species*	CI group3 (NF-independent)		++	
	*A. virginica* and two species**			+	
	*Stylosanthes hamata*		*Bradyrhizobium* SUTN9-2	−	This study
Phaseoloids	*Indigofera tinctoria*			+	
	*Desmodium tortuosum*			+	
Robinioids	*Lotus japonicus*		*Mesorhizobium loti* (*nifV* lacking strain)	−	(10)

++ in the dark gray cell, low plant growth with Δ*nifV* strains, similar to the non-inoculated control; + in light gray cells, low plant growth with Δ*nifV* strains, but better than the non-inoculated control; −, Δ*nifV* does not affect plant growth.

* *A. indica*, *A. scabra*, *A. sensitiva*, *A. deamii*, *A. denticulate*, and *A. tambacoudensis*.

** *A. pratensis* and *A. selloi*.
