# Supplementary material for: Homocitrate Synthase Genes of Two Wide-Host-Range Bradyrhizobium Strains are Differently Required for Symbiosis Depending on Host Plants
Source: Microbes Environ. 2019 Dec 27;34(4):393–401. doi: 10.1264/jsme2.ME19078 (PMC6934396; doi:10.1264/jsme2.ME19078)
Supplement: Supplementary file 1 [file 34_393_s1.pdf]

Supplemental Table S1. Bacterial strains carrying putative *nifV* gene in Fig. 1A.

Strain	Label in MicroScope	Gene product	Clade in Fig. 1A
Azotobacter vinelandii	AvCA_01640	nitrogen fixation homocitrate synthase	—
Mesorhizobium metallidurans STM2683	MESS2v1_980052	fragment of homocitrate synthase	I
M. amorphae CCNWGS0123	MEAMCv1_530020	homocitrate synthase	I
M. australicum WSM2073	MESAWv1_90056	homocitrate synthase	I
Sinorhizobium meliloti 4H41	AQWP_v1_80086	homocitrate synthase	I
Azorhizobium caulinodans ORS 571	AZC_3389	homocitrate synthase	—
***Bradyrhizobium* spp.**			
AT1	JXDL01_v1_12394	homocitrate synthase	II
BR 10245	LUUB01_v1_730060	homocitrate synthase	II
CCH5-F6	LSIC01_v1_480053	homocitrate synthase	II
cf659 CF659	FOQJ01_v1_180113	homocitrate synthase	II
Cp5.3	AUFA01_v1_450019	homocitrate synthase	II
DOA1	JXJM01_v1_40553	homocitrate synthase	II
DOA9	BRADOA9_v1_51508	homocitrate synthase	II
Ec3.3	AXAS01_v1_860039	homocitrate synthase	II
B. japonicum 22	AXVG01_v1_140448	homocitrate synthase	II
B. manausense BR3351	LJYG01_v1_190067	homocitrate synthase	II
B. neotropicale BR 10247	LSEF01_v1_260103	homocitrate synthase	II
S23321	S23_45990	putative homocitrate synthase	II
B. stylosanthis BR 446	LVEM01_v1_161248	homocitrate synthase	II
TSA1	LFJC01_v1_15448	homocitrate synthase	II
BR10280 p9-20	LWIG01_v1_560099	homocitrate synthase	II
BTAi1	BBta_5875	homocitrate synthase	III
B. oligotrophicum S58	S58_23130	homocitrate synthase	III
ORS278	BRADO5390	homocitrate synthase	III
ORS285	BRAD285_v2_1843	homocitrate synthase	III
ORS375	BRAO375v1_990107	homocitrate synthase	III
STM3809	BRAS3809v1_980003	homocitrate synthase	III
STM3843	BRAO3843v1_2960004	homocitrate synthase	III
ARR65	AWZU01_v1_550005	homocitrate synthase	—
Ai1a-2	AUEZ01_v1_1270014	homocitrate synthase	IV
B. elkanii WSM1741	AXAU01_v1_120180	homocitrate synthase	IV
B. elkanii WSM2783	AXAP01_v1_860003	homocitrate synthase	IV
B. jicamae PAC68	LLXZ01_v1_330023	homocitrate synthase	IV
B. lablabi CCBAU 23086	LLYB01_v1_1190019	homocitrate synthase	IV
LMTR 3	MAXC01_v1_520075	homocitrate synthase	IV
B. paxllaeri LMTR 21	MAXB01_v1_760050	homocitrate synthase	IV
B. retamae Ro19	LLYA01_v1_1890032	homocitrate synthase (fragment)	IV
Tv2a-2	AXAI01_v1_470008	homocitrate synthase	IV
B. valentinum LmjM3	LLXX01_v1_1670029	homocitrate synthase	IV
B. valentinum LmjM6	LLXY01_v1_110013	homocitrate synthase	IV
WSM2254	AXAB01_v1_150062	homocitrate synthase	IV
WSM3983	AXAY01_v1_230001	homocitrate synthase	IV
WSM3983	AXAY01_v1_290132	homocitrate synthase	IV
NAS96.2	LGHK01_v1_10011	homocitrate synthase (modular protein)	IV
B. icense LMTR 13	LMTR13_26450	homocitrate synthase	IV
CCGE-LA001	BCCGELA001_31050	homocitrate synthase	—
B. mercantei SEMIA 6399	MKFI01_v1_230945	homocitrate synthase	—
NAS96.2	LGHK01_v1_10081	homocitrate synthase	—
B. liaoningense CCNWSX0360	LUKO01_v1_2030012	homocitrate synthase (NifV)	—
B. embrapense SEMIA 6208	LFIP02_v1_60088	homocitrate synthase	V
UFLA 03-321	MPVQ01_v1_420055	homocitrate synthase	V
UFLA03-84	NSJY01_v1_120032	homocitrate synthase	V
B. viridifuturi SEMIA 690	LGTB01_v1_100079	homocitrate synthase	V
C9	NWTG01_v1_330116	homocitrate synthase	VI
B. elkanii 587	AJJK01_v1_23090039	homocitrate synthase	VI
B. elkanii BLY3-8	LWUI01_v1_180041	homocitrate synthase	VI
B. elkanii BLY6-1	LXEM01_v1_180011	homocitrate synthase	VI
B. elkanii CCBAU 05737	AJPV01_v1_4020045	homocitrate synthase	VI
B. elkanii CCBAU 43297	AJPW01_v1_3950013	homocitrate synthase	VI
B. elkanii USDA 3254	AXAH01_v1_260042	homocitrate synthase	VI
B. elkanii USDA 3259	AXAW01_v1_230039	homocitrate synthase	VI
B. elkanii USDA 76	ARAG_v1_51539	homocitrate synthase	VI
B. elkanii USDA 94	JAFC01_v1_1060046	homocitrate synthase	VI
B. pachyrhizi BR3262	LJYE_v1_810043	homocitrate synthase	VI
B. pachyrhizi PAC 48	LFIQ01_v1_660072	homocitrate synthase	VI
R5	FMZW01_v1_500036	homocitrate synthase	VI
WSM1743	AXAZ01_v1_20190	homocitrate synthase	—
B. arachidis LMG 26795	FPBQ01_v1_680095	homocitrate synthase	VII
err11 ERR11	FMAI01_v1_110319	homocitrate synthase	VII
genosp. SA-4 str. CB756	AXBC01_v1_60406	homocitrate synthase	VII
Ghvi	FOVU01_v1_350089	homocitrate synthase	VII
INPA54B	PGVG01_v1_610025	homocitrate synthase	VII
ORS3257	BRAD3257_v2_7801	homocitrate synthase	VII
ORS3409	BRAO3409v1_500145	homocitrate synthase	VII
B. ottawaense L2	NWTF01_v1_830037	homocitrate synthase	VII
Rc2d	FNCU01_v1_470212	homocitrate synthase	VII
Rc3b	FOUS01_v1_200023	homocitrate synthase	VII
SUTN92	SUTN92_v1_630027	homocitrate synthase	VII
USDA 3384	AXAD01_v1_90051	homocitrate synthase	VII
WSM2793	ARBF01_v1_200096	homocitrate synthase	VII
B. yuanmingense BR3267	LJYF_v1_290169	homocitrate synthase	VII
B. yuanmingense CCBAU 10071	FMAE01_v1_140007	homocitrate synthase NifV	VII
B. yuanmingense CCBAU 25021	AJQK01_v1_3620002	homocitrate synthase NifV	VII
BR10280 p9-20	LWIG01_v1_100281	homocitrate synthase	VII
B. yuanmingense CCBAU 35157	AJQL01_v1_4000104	homocitrate synthase	VII
